# Implementation of a nurse-delivered, community-based liver screening and assessment program for people with metabolic dysfunction-associated steatotic liver disease (LOCATE-NAFLD trial)

**DOI:** 10.1186/s12913-025-12580-5

**Published:** 2025-03-22

**Authors:** Michelle J. Allen, Ruth Tulleners, David Brain, James O’Beirne, Elizabeth E. Powell, Adrian Barnett, Patricia C. Valery, Sanjeewa Kularatna, Ingrid J. Hickman

**Affiliations:** 1 Australian Centre for Health Services Innovation and Centre for Healthcare Transformation, School of Public Health and Social Work, Faculty of Health, Queensland University of Technology, Brisbane, QLD Australia; 2 University of the Sunshine Coast, Maroochydore DC, QLD Australia; 3 Sunshine Coast University Hospital, Birtinya, QLD Australia; 4 Centre for Liver Disease Research, Translational Research Institute, Faculty of Medicine, The University of Queensland, Woolloongabba, QLD Australia; 5 Department of Gastroenterology and Hepatology, Princess Alexandra Hospital, Woolloongabba, QLD Australia; 6 QIMR Berghofer Medical Research Institute, Herston, QLD Australia; 7 Health Services and Systems Research, Duke - NUS Medical School, Singapore, Singapore; 8 Clinical Trials Capability, Centre for Clinical Research, The University of Queensland ULTRA Team, Herston, QLD 4006 Australia; 9 Department of Nutrition and Dietetics, Princess Alexandra Hospital, Woolloongabba, QLD Australia

**Keywords:** Non-alcoholic fatty liver disease, Metabolic dysfunction-associated steatotic liver disease, Community-based management, Randomised controlled trial, Implementation evaluation, RE-AIM framework, Acceptability

## Abstract

**Background:**

With the high burden of Metabolic dysfunction-associated steatotic liver disease (MASLD), (previously known as Non-Alcoholic Fatty Liver Disease - NAFLD) in the community, current models of care that require specialist review for disease risk stratification overwhelm hospital clinic capacity and create inefficiencies in care. The LOCal Assessment and Triage Evaluation of Non-Alcoholic Fatty Liver Disease (LOCATE-NAFLD) randomised trial compared usual care to a community-based nurse delivered liver risk assessment. This study evaluates the implementation strategy of the LOCATE model.

**Methods:**

The evaluation used mixed methods (quantitative trial data and qualitative framework analysis of semi-structured interviews) to explore the general practitioner (GP) and patient perspectives of acceptability (Acceptability Framework), and factors associated with reach, effectiveness, adoption, implementation, and maintenance (RE-AIM framework) of the LOCATE model of care.

**Results:**

The LOCATE model was considered highly acceptable by both patients and GPs. The model of care achieved appropriate reach across the participating health services, reaching high-risk patients faster than usual care and with predominantly positive patient experiences. A notable reduction in anxiety and stress was experienced in the intervention group due to the shorter waiting times between referral and assessment. There was an overall perception of confidence in nursing staff capability to perform the community-based screening and GPs indicated confidence in managing low-risk MASLD without the need for specialist review. Challenges to implementation, adoption and maintenance included variable prioritisation of liver disease assessment in complex cases, the need for further GP training in MASLD assessment and treatment pathways, available funding and referral pathways for community screening, and accessibility of effective diet and exercise professional support.

**Conclusion:**

Nurse delivered community-based liver screening is highly acceptable to GPs and patients and has shown to be an effective mechanism to identify high risk patients. Adoption and maintenance of the model of care faces significant challenges related to affordable access to screening, prioritisation of liver disease in complex patient cohorts, and unresolved difficulties in prescribing effective strategies for sustained lifestyle intervention in the primary care setting.

**Trial registration:**

The trial was registered on 30 January 2020 and can be found via Australian New Zealand Clinical Trials Registry (ANZCTR) – ACTRN12620000158965.

**Supplementary Information:**

The online version contains supplementary material available at 10.1186/s12913-025-12580-5.

## Contributions to the literature

- Community-based, nurse delivered liver screening is an acceptable mechanism to identify elevated risk of significant hepatic fibrosis and reduce wait times for specialist review in people with MASLD, with staff and patient experiences aligning positively to all components of the acceptability framework.
- Use of the RE-AIM framework enabled the identification and prediction of key challenges to the implementation, adoption, and long-term sustainability of the model of care beyond a trial setting.
- GPs highlighted factors to enhance uptake including promoting a value proposition to prioritise liver screening, including GP training in fibroscan interpretation, and lobbying for funding to improve affordable community access.

## Introduction

Metabolic dysfunction-associated steatotic liver disease (MASLD), (previously known as Non-Alcoholic Fatty Liver Disease – NAFLD) has global significance as the leading cause of chronic liver disease [1, 2]. MASLD is commonly accompanied by multiple comorbidities including hypertension, obesity, and type 2 diabetes [3, 4], and is associated with a reduced health-related quality of life. With prevalence increasing, the health system will incur greater costs associated with its diagnosis, management, and complications from disease progression [5, 6].

In Australia, usual care for people with MASLD who present in primary care settings with abnormal liver enzymes and/or steatosis on liver ultrasound, often involves referral to secondary care for confirmation of disease severity through an assessment of liver fibrosis by a liver specialist [7]. With such a high burden of MASLD in the community, this traditional model of care overwhelms hospital clinic capacity for screening and stratifying risk. The subsequent long waiting lists for risk stratification creates inefficiencies in care with low-risk patients engaging in unnecessary specialist assessments and high-risk patients potentially delayed in accessing treatment and surveillance.

A randomised trial comparing usual care to a new model of care with community-based fibrosis assessment (using vibration controlled transient elastography - Fibroscan), hypothesised to speed up the triage process, was undertaken called the LOCal Assessment and Triage Evaluation of Non-Alcoholic Fatty Liver Disease (LOCATE-NAFLD) trial [7]. Alongside clinical effectiveness measures and economic outcomes, this trial also measured implementation outcomes to understand the reach and acceptability of the model of care to both clinicians and patients, the barriers and facilitators of the implementation process, and factors that may influence sustainability and scale-up. A process evaluation whereby implementation outcomes are reviewed enables exploration of causal pathways, in relation to how and why an intervention does or does not work, within a given context [8]. Process evaluations can be particularly insightful when overall results are unexpected, in order to understand if there was an issue with the model of care itself, or in the way it was implemented, as both are considered important for implementation success [9, 10]. Process evaluations are also helpful to assess what is needed for the model of care to work in other settings if scale-up or spread is desired [8]. This study aims to present lessons from the implementation of the trial with a focus on patient and practitioner acceptability of the new model of care and factors which may influence the adoption and sustainability of the model outside the trial setting. Clinical effectiveness results and the economic analysis are reported separately [11].

## Methods

### LOCATE-NAFLD intervention (parent study)

The LOCal Assessment and Triage Evaluation of Non-Alcoholic Fatty Liver Disease (LOCATE-NAFLD) trial was a 1:1 parallel randomised trial comparing two models of care for MASLD – usual care versus Local Assessment and Triage Evaluation (LOCATE) model of care, with adults with suspected MASLD, undertaken in two health service areas in Queensland, Australia [7]. Of the 97 participants recruited from October 2020 to December 2022, 49 were randomised to receive the intervention [11].

**Fig. 1:**
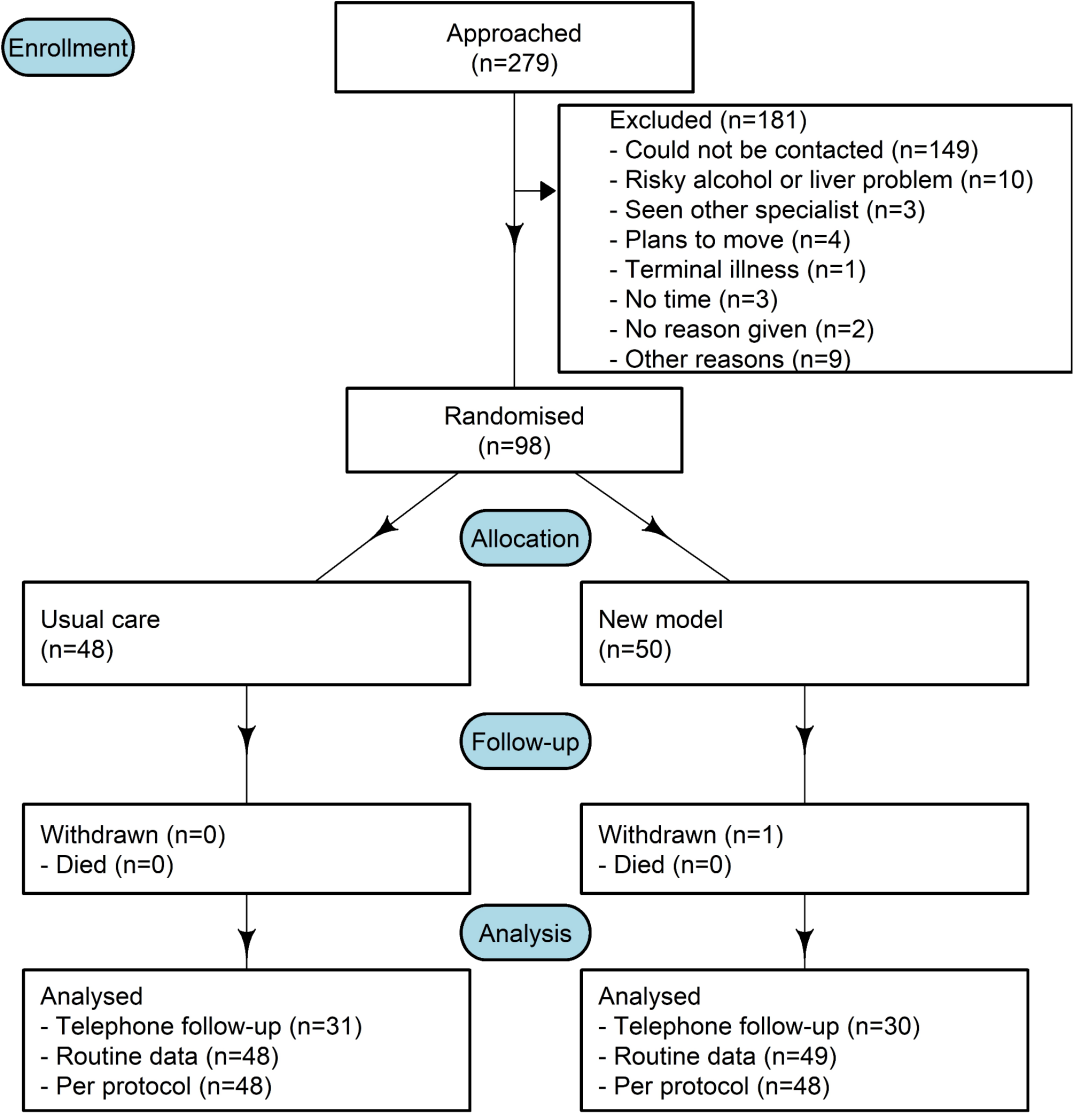
Recruitment and patient flow pathway for the LOCATE-NAFLD study

The flow diagram outlined in Fig. 1 describes the recruitment and patient flow pathway for the LOCATE-NAFLD study [7]. Those participants referred from general practice (GP) (primary care) and randomised to the new model of care were offered a community fibrosis assessment using fibroscan (measurement was taken using model Fibroscan Mini 430), undertaken by a specialist study nurse at a local community clinic. A hepatologist classified referrals into low or high risk for advanced disease based on the screening results. High risk patients were triaged to an earlier hospital (secondary care) appointment and if advanced fibrosis was confirmed, enrolled in hepatocellular carcinoma and variceal surveillance programs. Following review in clinic, low risk patients were referred back to their GP with an appropriate primary care treatment plan. For participants randomised to usual care, referral letters were triaged in the usual way with patients awaiting hepatologist review and fibroscan assessment at the hospital clinic. During the clinical trial period, patients received fibroscan assessment for free irrespective of the study arm.

The implementation evaluation objectives were to:

1. Explore the practitioner and patient acceptability of the LOCATE model of care using the Acceptability Framework.
2. Evaluate factors associated with implementation of the LOCATE model of care using the RE-AIM framework.

### Study design and theoretical framework

A mixed -methods evaluation, guided by the Reach, Effectiveness, Adoption, Implementation, Maintenance (RE-AIM) framework [12–14], was used to identify barriers and facilitators influencing individual, institutional and systemic factors related to the LOCATE model of care.

The RE-AIM framework has five domains: (1) Reach – number and spread of target population; (2) Effectiveness – impact of model of care on outcomes; (3) Adoption – uptake of ‘intervention agents’ (people who deliver the service) to undertake the model of care; (4) Implementation – the process of implementing the model of care, including fidelity; and (5) Maintenance – extent to which the model of care becomes ingrained in normal practice including sustainment and scalability [13].

In addition, the theoretical framework of acceptability was used [15]. This framework describes acceptability in terms of seven constructs: self-efficacy, affective attitude, perceived effectiveness, burden, intervention coherence, opportunity costs and ethicality [15]. This was incorporated into the analysis process to enable greater exploration of the GPs’ and patients’ experience and acceptability of the new model of care which is key to understanding its feasibility and sustainment of use.

### Data collection and analysis

#### Quantitative data

To provide data on recruitment and outcomes, we used the following data from the REDCap trial database: trial and evaluation recruitment rate, reasons for non-enrolment, and time from recruitment to scan and specialist review. To show the geographical reach, data were extracted from the GP referral letter (referral date, postcode of GP practice, and patient’s residential postcode). Postcodes were mapped to the Modified Monash Model (MMM) [16] suburb and locality classification that categorises participants’ place of residence as a city, rural, remote or very remote. We mapped patient and GP numbers by postcode using a choropleth map using the actual geographic boundaries and hexagonal map with equal sized boundaries as the results from small geographic areas are often lost when using geographic boundaries [17]. Animations that illustrate the links between the two map types are shown in Additional files 1 and 2. Descriptive analysis was undertaken in Excel to determine the reach of the study, and relevant implementation factors.

#### Qualitative data

Individual interviews were conducted via an online teleconferencing platform facilitated by an experienced mixed methods researcher (MJA) to explore GP and patient experience of the trial, and factors influencing uptake and acceptability. The target sample size was between 10 and 20 participants, representing a mix of referring GPs and patients who had experienced either the usual care or intervention arms. Trial participants were recruited for interviews during their 12-month follow-up phone call. GPs were provided with information on participating in interviews in the letter regarding trial eligibility. GPs who were interested in participating in an interview were asked to contact the research team for further information and were then approached by the team to arrange an interview. GP received a $100 gift card as compensation for their participation in the interview, with patients receiving a $20 gift card for their interview participation (in addition to compensation provided for participation in the trial).

The interview facilitator used a semi-structured interview guide (Additional file 3). This guide was based on the evaluation objectives and RE-AIM framework domains. The interview guide was designed to be flexible to allow exploration of implementation factors raised during the interview. All interviews were digitally recorded and transcribed verbatim using NVivo Pro (1.6.1). Themes were initially open coded using an inductive approach, then mapped to the RE-AIM and Acceptability frameworks using a deductive approach to framework analysis [18]. Framework analysis is a broad group of analyses that include both content and thematic analysis, where the data is reviewed for commonalities, differences, and relationships, at both a descriptive or explanatory level [18]. Framework analysis has seven key stages, all of which were followed including transcription of audio recordings, familiarisation with the data (repeated reading and listening), coding, developing a working framework for analysis, applying the analytical framework, charting data into the framework matrix, and interpreting the data [18]. Initial inductive coding was conducted separately by two qualitative researchers (IH and MJA), then codes and themes were discussed between three authors (IH, MJA, and RT) and mapped to both frameworks using an iterative process of constant comparison of codes and themes throughout. Themes and alignment with framework domains were discussed across the author group before finalisation. Illustrative quotes have been used, with consideration as to ensuring anonymity of participants, noting participant codes only (e.g. GP number 1 = GP1; Patient number 4 = PT4).

## Results

### Trial outcomes

Ninety-seven trial participants were recruited, with 49 (51%) randomised to receive the LOCATE intervention. The intervention demonstrated a decrease in the time to high-risk diagnosis, with a wide 95% credible interval for the hazard ratio, and the median reduction in ‘time to scan’ in the intervention was close to one year. Detailed trial results are reported in a separate manuscript [11].

### Implementation evaluation outcomes

Sixty-one participants were able to be contacted by phone for the 12-month trial follow-up and of these, 11 (18%) indicated an interest in participating in an interview for the purpose of the implementation evaluation, with a final total of 6 (usual care *n* = 2, intervention *n* = 4) completing an interview. There was representation from both trial sites.

Letters of invitation were sent to 116 referring GPs. Eight (7%) GPs expressed an interest in an interview, and 3 (3%) completed an interview for the purpose of the implementation evaluation. One GP participant provided care for a trial participant, whilst the other two GP participants had referred patients into the trial but were not subsequently enrolled.

Evaluation results are reported against the seven constructs of Acceptability, and the five RE-AIM framework domains.

#### Acceptability

Acceptability of the LOCATE model of care was mapped to six of the seven acceptability constructs (Table 1). Both GPs and patients understood how community screening could improve patient care through risk stratification (coherence), perceived community screening as an effective way to reduce wait times (perceived effectiveness), and were confident that a community nurse could deliver this care (ethicality). Participants expressed positive feelings about the intervention and the patient experience of the screening process (affective feelings), as well as the non-invasive and easily accessible nature of community screening (patient burden). GPs were confident in managing low-risk MASLD in the community but noted additional education would likely be required for interpretation (self-efficacy). Participants did not articulate any trade-offs in relation to the screening process (opportunity cost) but did highlight competing priorities in relation to subsequent lifestyle modification supports. This demonstrates that the acceptability of the LOCATE model is multi-faceted and therefore more likely to have broad applicability.

**Table 1:** Exemplar quotes mapped to the acceptability framework constructs

Acceptability Construct	Exemplar Quotes	Summary
Coherence (understanding how the intervention works)	*PT6: “Because I did realise that a lot of specialists do take some time to get in*,* and I thought*,* well*,* if this is going to be a way to save people waiting - then yeah (I’ll participate).”* *GP1: “I have to send some patients to hospital just because we can’t check them without a Fibroscan.”*	There was broad understanding from both practitioners and patients about how community screening may improve efficiencies in access to risk stratification screening
Self-efficacy (personal ability to do it)	*GP2: “Quite confident if deemed as low risk. (We are) well set up for NAFLD care. All doctors (in our group) are well versed in NAFLD treatment*,* plus we have some staff who can do motivational interviewing.”*	Practitioners felt confident to manage low risk MASLD without specialist review. There was variable practitioner confidence in understanding and interpreting Fibroscan results with identified need for workforce training
Affective attitude (Feelings about the intervention)	*GP1: “So*,* they can be waiting months and months*,* sometimes years to see a Hepatologist… if we have little bit of the Fibroscan available*,* I suppose we can even stop the moderate fibrosis patient going to hospital… for me*,* it’s going to be very helpful to have the Fibroscan available in the community.”*	There were overall positive feelings about the intervention, as well as positive experience of receiving the intervention
Perceived effectiveness (does it serve its purpose)	*GP1: “So this project will stop the people on the waiting list if people who don’t need to be on the waiting list… it would also decrease the burden on the hospital system*,* and it’s also going to be decreasing waiting time for the patients.”*	There was overall perceived effectiveness of community-based screening to reduce waiting times for screening from both practitioners and patients
Burden (Effort to participate)	*GP2: “Easy access to the facilities where the Fibroscan is… like radiology centres where they probably go normally for scans….”* *PT3: “Yeah*,* that was fine. It wasn’t invasive or anything.”*	The burden of community-based screening was considered low
Opportunity costs (what is given up to participate)	*No related quotes*	There were no specific opportunity costs related to community based screening highlighted.
Ethicality (Good fit with values)	*PT6: “So*,* I think in future… even if it wasn’t a study and (I was) just told yes*,* a nurse can do it for you*,* I wouldn’t have an issue with it…I didn’t feel I had to speak to the specialists. I found all the nursing staff to be quite capable.”*	There was an overall acceptance that trained nurses in the community were appropriate to perform screening

#### Reach

During the trial recruitment period, 279 potentially eligible participants were identified and invited to participate. One hundred and twenty-nine invited participants contacted the study nurse to express their interest in participating. The remainder did not respond to the invitation, despite a range of contact and information methods being used, including personalised letters, a recruitment video, and phone calls.

**Fig. 2:**
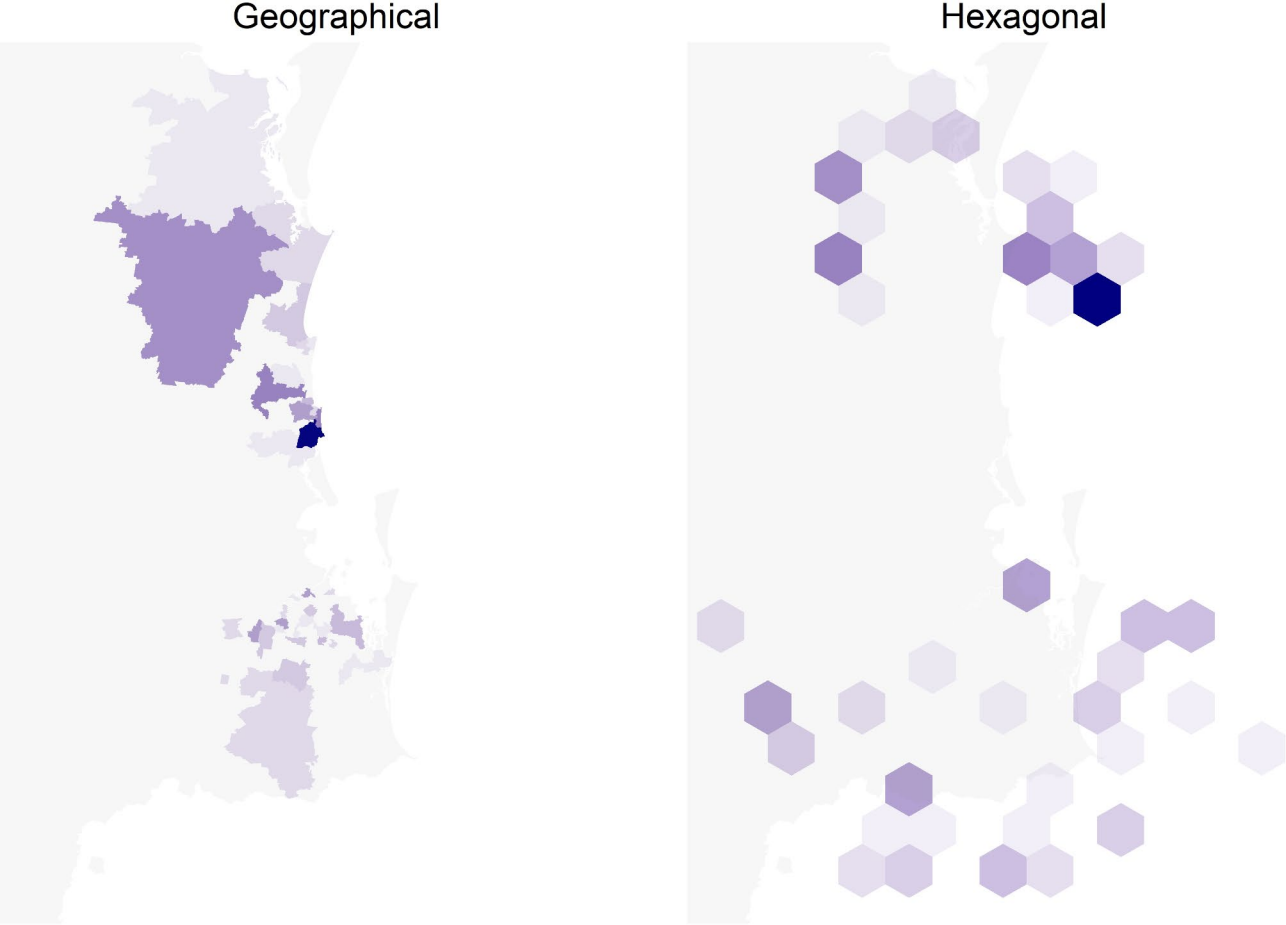
Maps of the south-east Queensland area with the number of patients by postcode using geographical (**a**) and hexagonal (**b**) boundaries. The hexagonal boundaries use equally sized postcodes as an alternative to the geographic boundaries. An animation in the Additional file 1 illustrates how the geographical and hexagonal boundaries are linked

**Fig. 3:**
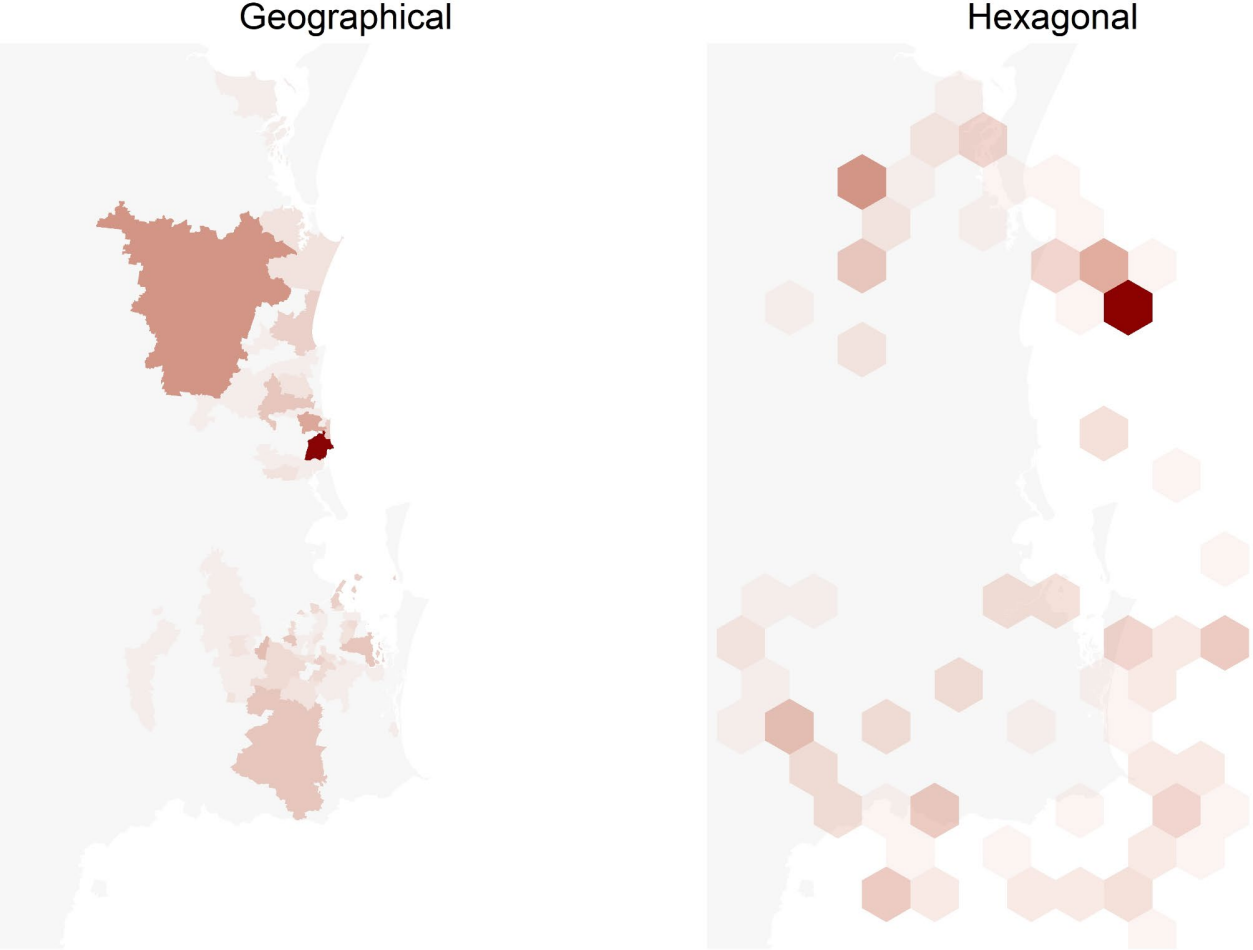
Maps of the south-east Queensland area with the number of GPs by postcode using geographical (**a**) and hexagonal (**b**) boundaries. The hexagonal boundaries use equally sized postcodes as an alternative to the geographic boundaries. An animation in Additional file 2 illustrates how the geographical and hexagonal boundaries are linked

Participants in the LOCATE-NAFLD trial resided in metropolitan (*n* = 81, 84% MM1), regional (*n* = 5, 5% MM2 and *n* = 4, 4% MM3) or in rural/remote (*n* = 2, 2% MM4 and *n* = 5, 5% MM5) locations. The maps in Figs. 2a and b and 3a and b show two concentrations of patients and GPs respectively, one to the north of Brisbane around the Sunshine Coast University hospital and one to the south of Brisbane around the Logan hospital.

Most participating GP postcodes were metropolitan (*n* = 89, 80% MM1). However, there was representation from GPs in regional post codes (*n* = 9, 8% in MM2 and *n* = 8, 7% in MM3) and more remote areas (*n* = 2, 2% MM4 and *n* = 3, 3% MM5). There were 20 GPs (18%) that referred more than once. Given the focus of the trial was on two key health service areas that are largely based within metropolitan and inner regional areas, this means that the reach of this intervention captured patients beyond the expected post codes.

In addition to geographical reach, the reach of the intervention to higher risk patients (defined as transient elastography ≥ 8.0 kPa) was also assessed in relation to time to receive a scan. The LOCATE model of care was able to reach high risk patients faster than usual care due to quicker access to scans with median time to scan for high-risk patients 10 days vs. 296 days respectively from time of randomisation. In the intervention arm, all patients received a scan and 100% of high-risk patients saw a specialist with a median time of 110 days. By the end of the trial period, the usual care model had not reached 11 (23%) patients for scanning. Of those scanned and identified as high risk in the usual care model, the median time to see a specialist was 355 days.

#### Implementation effectiveness

Implementation effectiveness includes patient and GP reported impacts of participation in the trial and were expressed in three key ways– anxiety reduction, system burden reduction, and effective delivery of community-based scans by nurses. Participants identified that their referral for assessment of liver disease severity was associated with significant anxiety and stress, and those worries were alleviated more promptly in the intervention group than the usual care group, due to the shorter wait time for screening.

In relation to effectiveness of the intervention, GPs reported their perception that community screening would reduce wait times and the subsequent burden on the hospital system. In terms of effectiveness of nurse-delivered community screening, patients highlighted the excellent communication skills and capability of nursing staff including their ability to explain the fibroscan results in a coherent way. This aligns with patient-reported acceptability and confidence in nurse-delivered screening. Exemplar quotes about effectiveness from GP and patient participants are shown in Table 2.

**Table 2:** Exemplar quotes mapped to the RE-AIM domains effectiveness, adoption, implementation and maintenance

**(Implementation) Effectiveness domain**	**Summary**
*PT3 (NMC) “To have that done*,* very quick(ly)*,* it was like a relief for me because I was very*,* very stressed”* *PT6 (NMC) “(I was) just really in limbo as to what I was going to do and then it was good to get the phone call to say*,* hey you can jump the queue by not seeing specialist but a nurse. So that was good reassurance… definitely good to speed up the process because when you hear something like that you freak out because you don’t understand it… it would be annoying now if I was still waiting to see a specialist. At least now I know that I have had improvement and that it’s not something to be worried about. Just need to make some lifestyle changes.”*	Earlier access to screening resulted in a reduction in stress and anxiety for patients
*GP1 “So*,* they can be waiting months and months*,* sometimes years to see a Hepatologist… if we have little bit of the Fibroscan available*,* I suppose we can even stop the moderate fibrosis patient going to hospital… for me*,* it’s going to be very helpful to have the Fibroscan available in the community.”*	LOCATE model was perceived to be effective at improving access for patients and reducing hospital burden
*PT3 (NMC) “She just explain(ed) everything to me…show(ed) me the nearest screen and I saw everything there.”* *PT2 (NMC) “The lady there was wonderful. Really went and explained what she was doing. In a way that I think lay people like myself would understand.”* *PT5 (UC) “(it) would be nice to know exactly what it was*,* straight away… even within a fortnight*,* you know… I think I called to find out the information. Yeah. And they said that it hadn’t been reported on*,* and this is like four months since I’d had it. And it still hadn’t been reported on. So that’s pretty poor.”*	Nurses undertaking the LOCATE model were effective at communicating the test process and subsequent results Communication delays were experienced in the usual care arm.
**Adoption domain**	**Summary**
*GP3 “I reckon I would diagnose NAFLD– once a week. Super common. So*,* a typical scenario would be maybe someone I send for check-up blood tests and their liver function tests are abnormal… and by and large the most common diagnosis is NAFLD… I just think it’s such an important topic… thankfully*,* most people with fatty liver don’t go on to get cirrhosis*,* but the rates of cirrhosis are increasing*,* as is patients having to have a transplant because of fatty liver and cirrhosis they cascade. So I just think it’s such an important area that we shouldn’t be brushing over and saying just fatty liver.”*	GPs regularly manage patients with potential liver conditions and understand the need for better access to liver screening assessments
*GP1 “I think for me*,* it’s going to be very*,* very helpful. But to be honest*,* when I came to speak to most of my colleagues…I’m talking about the general practitioners*,* they’re not very familiar and they don’t want to invest their time reading up… They find it easier just to refer the patient… If you are talking about the whole of the general practitioners*,* my understanding is that if you just make it (Fibroscan) available*,* most of the doctors they’re not going to use it because they don’t even*,* they don’t know how to utilize it*,* don’t know how to interpret it… they (need to) get better educated… I suppose even one page of information is enough to them to understand*,* accept it. I think that could stop heaps of patient going onto the waiting list.”* *GP2 “Getting a score is good*,* but further interpretation would be useful. A bit more information on what each of the scores means… How long do they have to “sort out” their lifestyle before they get into the next (risk) zone*,* etc.”*	Further education for GPs is needed to support their referral to, and interpretation and use of screening results for broad adoption of the LOCATE model
*GP1 “I have to send some patients to hospital just because we can’t check them without a Fibroscan.”* *GP3 “I think I would be incredibly valuable to be able to refer for Fibroscan directly because that’s (got) the best evidence… the main reason I refer to hospitals… if it’s purely NAFLD*,* then it’s really to get that opinion - Is there cirrhosis or not? The other time that I refer people is if I’m not sure NAFLD is the only thing going on… If it’s purely NAFLD then the main thing I am after is the Fibroscan.”*	GPs highlighted a willingness to adopt the LOCATE model due to lack of access to diagnostic tools for MASLD outside the specialist setting
*GP3 “I’m pretty confident*,* actually*,* unless there was something that didn’t add up… but really other than something weird going on*,* I would (be) quite confident managing myself*,* yeah.”* *GP1 “I think I am pretty alright with that. From my knowledge of fatty liver disease*,* there is no particular pharmacological intervention*,* so at the end*,* it all comes to down to risk factor modification… All of these things we can do in primary care… I’m pretty confident that up to moderate fibrosis can be treated in the community… these are all things we can do by ourselves.”*	GPs are confident addressing low risk MASLD in the community without specialist review
**Implementation domain**	**Summary**
*GP3 “Making sure its accessible to patients. I’ve got a lot of patients*,* you know*,* they wouldn’t drive to the north side of Brisbane*,* for example*,* for a test. It has to be*,* you know*,* on the right side of town*,* so to speak. And I think that’s important. Even simple things like parking. Can they get there in park or is there a fee for parking or is it in a major centre?…then I know the funding and the cost*,* if any*,* to the patient*,* I think is another barrier to customer.”* *GP2 “Easy access to the facilities where the Fibroscan is… like radiology centres where they probably go normally for scans.”*	GPs highlighted the importance of suitable locations and the need for access to community screening at low cost for patients.
*PT2 (NMC) “I went and had that scan*,* the non-invasive scan. Look*,* I just remember [family member] having to go and having biopsies… but sometimes it’s quite invasive… Whereas having that scan was just great… It was perfect. It was just the local hospital. And you*,* you know*,* you just sat and waited your turn*,* like a doctor’s surgery… (but) it would be great if you could get in sooner…”* *PT4 (NMC) “Yeah*,* that was that was fine. It wasn’t invasive or anything.”* *PT1 (UC) “I jumped on the train. I didn’t have to walk far*,* they saw me straight away*,* they were magical…. The only thing I did say is why did I have to wait to go to the hospital when I could have probably had it done elsewhere?”* *PT6 (NMC) “It hasn’t been an issue for me. It’s fine. And even if I didn’t have a car*,* there is public transport and everything*,* so that’s fine. It was easy to find*,* and everyone’s been friendly and that’s good.”*	Patients highlighted the non-invasive nature of the screening test and the relative ease of access but flagged timeliness and location as key access considerations.
*PT1 (UC) “Oh*,* they were lovely over at the [hospital]… They were really good. Treated me absolutely lovely”* *PT4 (NMC) “Oh*,* good*,* good. Actually*,* the nurse was amazing*,* to be honest…. No*,* just positive feedback… I’m happy with the whole overall process - from seeing*,* from my GP to you guys*,* and go back to my GP*,* and everything has been very smooth.”* *PT6 (NMC) “I did find that last nurse was quite quick. I did feel that in the last appointment that I had she was time sensitive. Like*,* you could tell she was just in a hurry with me. That wasn’t a good feeling… I felt like I was just a number to her.”*	Patients noted predominantly positive interpersonal experiences with the nursing staff performing the screening assessments, but this was not universal.
*PT4 (NMC) “This is a kind of normal for you. I don’t drink. I usually have a good diet. I exercise every day. So*,* we have been checking up every six months and having a blood test since then. And then the numbers haven’t changed at all yet…. He’s actually pretty happy with my normal life.”* *PT1 (UC) “I don’t take any medication… I’m not on anything… So*,* you know I’m pretty healthy… not like all the pills my friends are on.”* *PT6 (NMC) “I actually do lots of exercise*,* so I don’t really have an issue in the exercise area. Obviously*,* my diet is the issue*,* so I could really use some advice on dietary.”*	There was a range of awareness and experiences related to MASLD diagnoses and the understanding of the relative contribution of lifestyle factors to liver disease.
**Maintenance domain**	**Summary**
*GP1 “Number 1. Fibroscan available for some patients on Medicare so they don’t have to pay out of pocket*,* 2. Able to be done without patients going on the waiting list*,* and 3. Just be able to refer to Fibroscan (performed) by a nurse*,* not have to go to hospital… (but) that’s going to be a big job to get that on Medicare through the Federal government. Maybe just through the state level… No need to see Hepatology… like fast-track fibro-scan.”* *GP3 “Previously we had people come and do some lunch time meetings… whoever championed for hepatitis B*,* you know*,* offering free Fibroscans for people with hepatitis B. Or*,* just knowing that it (Fibroscan) was even an option… the other way is through the general primary health networks. And certainly*,* a lot of us subscribe to the weekly newsletter from them. And that goes is not only to the GP who prescribe*,* subscribes*,* but also to practice nurses. And a lot of practice nurses are really good resources in terms of what’s available in the community and they can share which can be beneficial to everyone the practice. I think it is a really good way of reaching a lot of people.”*	A Medicare rebate and GP education were recommended for sustainability of any future scale-up of the LOCATE model
*GP3 “The usual barriers to lifestyle management*,* unfortunately*,* as it’s hard. It’s not as simple as just taking a tablet. What we all want things that we can fix easily. And you know*,* the idea of taking a tablet*,* I can understand that - I have this tablet*,* I understand that my blood pressure or my Diabetes or whatever it is*,* it’s fixed. And the fact that there is no option like that for NAFLD*,* I think it makes it really tricky because it is hard to exercise*,* and it is hard to lose weight. And so those things*,* I think*,* you know*,* that’s almost universally difficult.”* *GP3 “I suppose I work in an area that is mixed SES (socioeconomic status)*,* so for some people money is not a problem*,* whereas for other people might struggle to see a dietitian or see an exercise physiologist. And there’s things we can do to try to help*,* such as their care plan*,* where they can access allied health professionals*,* but there might still be a gap*,* say on top of that. If you’re really struggling to pay the bills an extra $30*,* you know*,* to see the dietitian*,* is a lot of money… the other thing that people have been feeding back to me particularly recently is just the cost of living and how expensive groceries are… I think financial limitations is certainly a big thing as well when it comes to making those lifestyle changes”*	GPs highlighted multiple barriers to subsequent lifestyle modification once screening results had been received by patients, such as cost of living pressures.
*PT2 (NMC) “When I was younger… I was exercising five*,* six times a week… But I struggle with it now when I’m trying to work full time and manage a home*,* my husband often works away so it’s like just me delivering boys in different directions*,* and it’s really hard.”* *PT6 (NMC) “I am allowed to get some (exercise) support and dietary (advice)*,* however*,* because I can’t get a whole Tuesday off… (I’m) on the wait list. So that’s kind of been disappointing… I’ve got an appointment*,* which I just confirmed today in [3-months’ time]… I gave up and found my own dietitian. My GP is not really*,* well*,* she’s in a hurry all the time… I feel going back to her would be a waste of time.”* *PT5 (UC) “I’ve just dropped one day of work a week in the last couple of months… so that I can accommodate the appointments… Again*,* they haven’t really given me any advice other than other than the Mediterranean diet… I kind of think this day off will help me with losing weight.”*	Patients noted both individual and system level barriers to lifestyle modification such as competing work and family commitments, as well as challenges accessing publicly funded services

*UC* Usual care, *NMC* New Model of Care (Intervention)

#### Adoption

The adoption domain of the RE-AIM framework focuses on the “intervention agents,” which (outside of a trial setting) would be GPs who would refer to a community fibrosis scanning service and drive uptake of the service model. As such, we have analysed adoption in reference to GP awareness and understanding of MASLD and fibrosis screening, their ability to interpret the fibroscan results, and their confidence managing low-risk MASLD in the community without specialist review.

GPs all noted an awareness of the high prevalence of MASLD in the community and the need for service improvement due to the long waiting lists for specialist Hepatology clinics. However, GPs flagged that understanding and interpreting fibroscan assessments was not common knowledge across the workforce, and further education may be needed before scaling this model of care. GPs also suggested that referring patients with MASLD to hospital clinics is often to access fibrosis assessment to enable risk stratification (high or low risk) and is likely to continue to be the main driver for engaging with the LOCATE screening model if it becomes available. All GPs highlighted their confidence in treating low risk MASLD in community settings without specialist review and therefore referral to the hospital system if screening results showed low risk was considered unlikely. Exemplar quotes about adoption from GP participants are shown in Table 2.

#### Implementation

The trial fidelity has been reported elsewhere [11] noting that study protocols were well adhered to. This evaluation focused on the reasons for non-participation, as well as the patient and GP experience of the new model of care.

In addition to those who did not respond to the invitation (*n* = 100), those who responded but did not participate (*n* = 50) provided reasons such as: Being ‘happy to wait’ for a specialist appointment outside of trial enrolment (*n* = 12, 24%); Difficulty attending community screening due to distance (e.g. living far away) or difficulties accessing transport (*n* = 6, 12%); Health reasons (*n* = 5, 10%); Too busy (*n* = 5, 10%); Work commitments (*n* = 3, 6%); No reason given (*n* = 13, 26%); and Other (*n* = 6, 12%) such as language barriers or high self-reported alcohol use. GPs identified potential implementation challenges to community-based screening if it were to be used beyond the trial, such as accessibility in relation to location and cost.

The experience of receiving the screening test were reported positively regardless of whether patients were seen in the community or at the hospital, including the non-invasive nature of the scan. However, patients in the usual care group who received fibroscan screening at a hospital location indicated an earlier appointment or a more convenient location would have been preferable. Most patients irrespective of group allocation described having a positive experience with the nursing staff performing the fibroscan, but this was not universal.

Across the patient group there was variable understanding about the relationship between diet and exercise and liver disease and there appeared to be a disconnect or confusion in some patients about the contribution of their own diet and exercise habits to their risk of liver disease, which was not resolved with fibroscan assessments. Exemplar quotes about implementation from GP and patient participants are shown in Table 2.

#### Maintenance

Critical system level factors that would need to be facilitated for scalability and subsequent sustainability of the LOCATE model were reported to include the availability of a Medicare rebate to support affordable and equitable access, and education for GPs to encourage appropriate access to and use of fibroscan assessments as part of MASLD diagnosis and management.

Whilst integration into routine care was not a part of the trial, we have reported the current and future barriers to accessing community-based fibrosis assessment, the challenges with accessing ongoing lifestyle modification, and other systemic issues under the maintenance domain, as these will be of value to policy makers in relation to long-term sustainability and scale-up. GPs highlighted the inherent challenges of lifestyle modification, as well as additional barriers due to the rise in cost of living and competing work and family time commitments. Patients raised further challenges related to accessing publicly funded screening and specialist services such as restricted days and times, needing to take time off work to attend these services, lack of personalised information, or lack of GP support. Exemplar quotes about maintenance from GP and patient participants are shown in Table 2.

## Discussion

Implementation of a nurse delivered community-based liver assessment for people with MASLD was considered highly acceptable by both patients and GPs. The model of care achieved appropriate reach across the participating health services, reaching high-risk patients faster than usual care (noting wide 95% credible interval for hazard ratio) and with predominantly positive patient experiences. There was a reported reduction in anxiety and stress related to shorter waiting times between referral and assessment in the intervention group who participated in the interviews, which was an unexpected benefit of the new model of care. Those participating in the interviews reported an overall perception of confidence in nursing staff capability to perform the community-based screening, and confidence in GP’s managing low risk MASLD without the need for specialist review. Challenges to implementation, adoption and long-term sustainability beyond a trial setting included variable prioritisation of liver disease assessment by GPs in complex cases, the need for further GP training in MASLD assessment and treatment pathways including fibroscan interpretation, available funding and referral pathways for community screening, and accessibility of effective professional diet and exercise support.

Overcoming a lack of awareness of liver disease in the community has been identified as a priority for screening programs [19, 20]. In addition to accuracy of assessments, factors related to ease and convenience of access, positive interactions between consumer and health care workers, and system level issues such as cost and wait time have all been highlighted as important factors for adoption of screening [19]. This study supports these findings with similar challenges to uptake and maintenance identified by GPs and patients.

The reported stress and anxiety experienced whilst waiting for risk assessment, and the subsequent faster resolution of those feelings, was a positive consequence of the intervention which was highlighted by both patients and GPs. These findings are similar to a recent qualitative study of people with risk factors for liver disease, including high alcohol intake, who received fibroscan screening in primary care, where patients reported a sense of relief post-screening for those with a lower risk level [21].

The overall broad acceptance of community-based screening in this study has been shown in other liver diseases where despite often low baseline knowledge of liver disease, patients report a positive experience with, and an acceptance for, fibroscan screening in primary care settings [21]. In an Australian study of people with Hepatitis C infection considering treatment, 95% of participants considered community based fibroscan as a ‘very acceptable’ method of liver assessment [22]. Acceptability results from these studies and the reduction in wait time supports the introduction of community screening for liver disease.

GPs noted several challenges related to adoption, implementation, and maintenance of the new model of care. The need for GP education on the purpose and value of liver assessment for MASLD and the interpretation of fibroscan reports as well as greater affordable access to fibrosis screening in the community as part of primary care pathways were identified. This aligns with global research priorities in MASLD that identified the need for awareness raising and education both for better definition and implementation of models of care [20, 23]The provision of effective training to GPs remains challenging and not limited to models of care for liver disease. A recent study implementing online training to assist GPs to deliver evidence-based management plans for osteoarthritis, including diet and exercise prescription, experienced a training module completion rate of between 15 and 53% of GPs enrolled [24]. Further, a multi-country study into the use of non-invasive testing for diagnosis and risk stratification in MASLD, similarly demonstrated limited knowledge and awareness among GPs and unclear referral pathways among the key reasons hindering uptake [23]. Targeted education campaigns to address the knowledge practice gap in MASLD management are warranted prior to attempts at scaling this community screening initiative.

Whilst a lack of knowledge and skills about implementing MASLD guidelines are a key barrier to adoption, other important barriers also play a role. These include primary care environmental context and resources, and frequent need to prioritise other aspects of treatment goals. In addition, a lack of effective strategies for behavioural regulation related to diet and exercise interventions can influence prioritisation of management plans [25]. In a recent Australian study that assessed the barriers and facilitators of implementing a MASLD pathway within a specialist GP diabetes clinic, GPs were confident in managing MASLD in primary care, and despite agreeing that community fibrosis screening may improve patient care, this was often not prioritised as it was deemed unlikely to change treatment course [26]. Across other settings, GPs have expressed confidence in managing MASLD without an assessment of disease severity [27, 28]. The GPs in this study expressed confidence in managing low risk MASLD without referral to a specialist and that the driving incentive to adopt community fibrosis screening was not related to improved confidence to manage patients, but rather a better ability to appropriately triage referrals of high-risk patients to specialist centres.

Lifestyle modifications in relation to improving diet quality, increasing exercise, and reducing alcohol consumption are considered the cornerstone to effective MASLD treatment [29–31]. However, creating and maintaining these modifications is not without its challenges, including GP confidence and time to prescribe lifestyle modifications [32–34]. Receiving fibrosis assessment in either model of care did not appear to overcome several recognised challenges related to diet and exercise change such as competing priorities of work and family, difficulty accessing services, cost, and the need for more personalised information and support. These challenges have been found across multiple studies relating to lifestyle modification for chronic disease management [29, 35–42]. One longitudinal interventional study implementing lifestyle modification for Type 2 Diabetes assessed the motivation and barriers of participants and found similar challenges such as time, work, and family pressures; sustaining energy and focus; accessibility of support services; and limited disease-specific knowledge [37]. Further, a meta-analysis on Type 2 Diabetes care demonstrated that lifestyle modifications are rarely sustained post-trial [38]. These studies demonstrate that once screening takes place and a diagnosis has been made, such as was trialled in the LOCATE-NAFLD study, consideration is needed around effective support for people with MASLD to make and maintain lifestyle modifications. Despite newly developed pharmaceuticals showing promise as an aid to weight loss, there remains low GP awareness or confidence in appropriate prescription as a complement to diet and exercise changes for MASLD management [43]. Hybrid effectiveness-implementation studies are needed to test treatment strategies incorporating patient centred dietary approaches [44], targeted exercise prescriptions [31] and promising eHealth implementation strategies [45] in a primary care setting.

There are some limitations to this evaluation that must be considered. Although a diversity of experiences were captured, the low response rate of patient and GP participants for the post-trial interview potentially reduces the generalisability of the findings and means we may have missed some important viewpoints including those of the trained nurses who performed the fibroscan assessments. The responses are relevant only to the local health context of Australia which limits generalisability of the results more broadly. The study was conducted during public health restrictions related to the COVID-19 pandemic which impacted recruitment and access to health care services and may have influenced the perceptions of prioritisation for health screening practices during this time.

## Conclusion

Nurse delivered community-based liver screening is highly acceptable to GPs and patients and has shown to be an effective mechanism to identify high risk patients. There remain significant challenges with maintaining adoption of the model in practice related to affordable access to screening equipment, prioritisation of liver disease in complex patient cohorts and unresolved difficulties in prescribing effective strategies for sustained lifestyle intervention in the primary care setting.

## Supplementary Information

Additional file 1. GP reach data animation. Animation that shows the data that collected about where referring GPs were located switching between geographical data and hexagonally displayed data.

Additional file 2. Patient reach data animation. Animation that shows the data that collected about where participating patients were located switching between geographical data and hexagonally displayed data.

Additional file 3. Interview guide. This is the semi-structured guide used for interviewing GPs and patients.

## Data Availability

Data collected for this process evaluation has either been provided within the manuscript or supplementary information, or is available on reasonable request with ethical approval, except for raw audio files and transcripts as these are potentially re-identifiable.

## Abbreviations

COVID-19: Coronavirus disease 2019
GP: General Practitioner
HREC: Human Research Ethics Committee
kPa: Kilopascal
LOCATE: LOCal Assessment and Triage Evaluation (name of model of care)
LOCATE-NAFLD: LOCal Assessment and Triage Evaluation of Non-Alcoholic Fatty Liver Disease (name of trial)
MMM: Modified Monash Model (NB: may be expressed as MM1 through to MM5)
NAFLD: Non-Alcoholic Fatty Liver Disease
NMC: New Model of Care (Intervention arm)
RE-AIM: Reach Effectiveness Adoption Implementation Maintenance Framework
MASLD: Metabolic dysfunction-associated steatotic liver disease
UC: Usual care arm
