# Supplementary figures and images for: Implementation of a nurse-delivered, community-based liver screening and assessment program for people with metabolic dysfunction-associated steatotic liver disease (LOCATE-NAFLD trial)
Source: BMC Health Serv Res. 2025 Mar 22;25:421. doi: 10.1186/s12913-025-12580-5 (PMC11929169; doi:10.1186/s12913-025-12580-5)

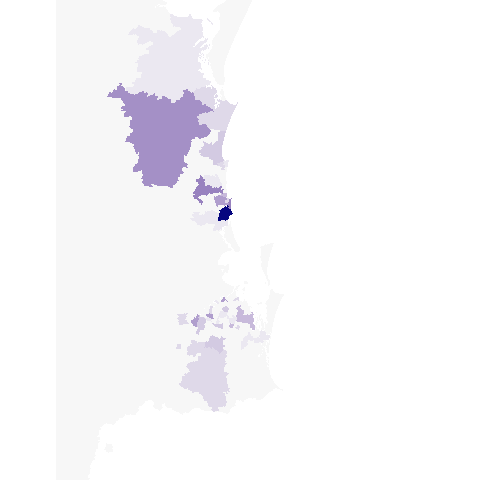

Supplement: Supplementary file 1 — Additional file 1. GP reach data animation. Animation that shows the data that collected about where referring GPs were located switching between geographical data and hexagonally displayed data. [file 12913_2025_12580_MOESM1_ESM.gif]

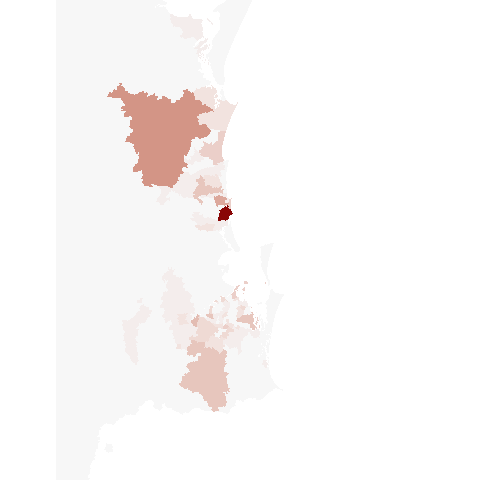

Supplement: Supplementary file 2 — Additional file 2. Patient reach data animation. Animation that shows the data that collected about where participating patients were located switching between geographical data and hexagonally displayed data. [file 12913_2025_12580_MOESM2_ESM.gif]
