# Supplementary material for: Implementation of a nurse-delivered, community-based liver screening and assessment program for people with metabolic dysfunction-associated steatotic liver disease (LOCATE-NAFLD trial)
Source: BMC Health Serv Res. 2025 Mar 22;25:421. doi: 10.1186/s12913-025-12580-5 (PMC11929169; doi:10.1186/s12913-025-12580-5)
Supplement: Supplementary file 3 — Additional file 3. Interview guide. This is the semi-structured guide used for interviewing GPs and patients. [file 12913_2025_12580_MOESM3_ESM.docx]

| **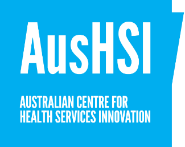** | 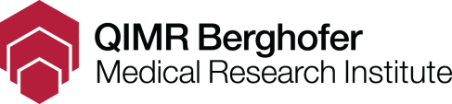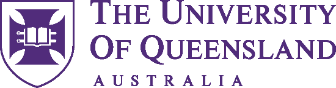 | 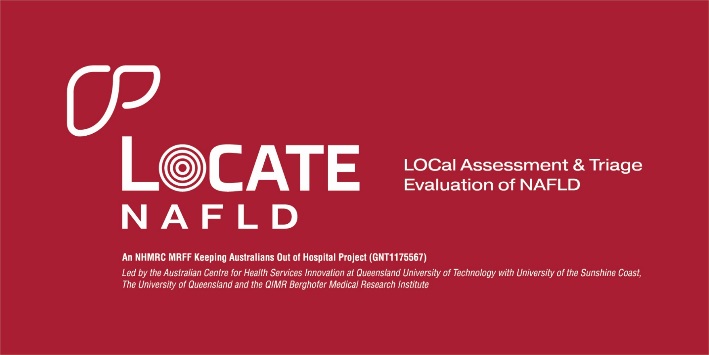 |
| --- | --- | --- |

**LOCATE-NAFLD Semi-Structured GP Health Professions and Patient Interviews: Outline**

**Aims:**

- To determine the factors associated with implementation of the model of care including a) individual factors (awareness, knowledge, skills, acceptance); b) institutional factors (practice management barriers and enablers); and c) systemic factors (communication processes, referral processes, triage workflow, waiting lists etc)
- To explore the Health Professional and Patient experience with the model of care including acceptability of community based non-specialist screening and community-based care for lower risk conditions

**Structure:**

The content of the group and individual interviews will be based on the RE-AIM model. Questions will be tailored to reflect the timing of the interviews in relation to the trial implementation phases. The interviews will be semi-structured, with open-ended questions and adaptable questions to reflect the responses being provided.

**Content:**

Review aims of session, participant information sheet, particularly confidentiality, consent, privacy and right not to participate. Collect signed consent forms.

*Note: Not all sections may be covered in each session, depending on participant numbers, roles, flow of discussion, time constraints and particular trial phase.*

|  | | **Timing** | |
| --- | --- | --- | --- |
| **Objective** | **Question content topics** | **During Trial** | **Post-Trial** |
| Assess the awareness of model of care across a cohort of referring GPs | Awareness of the LOCATE study |  |  |
| To explore the health professional and patient experience, with the model of care, including perceptions of experience and the acceptability of community based non-specialist screening and community based care for lower risk conditions | Experience/perceptions of community-based screening and treatment options for NAFLD |  |  |
| To determine the factors associated with implementation of the model of care, including:   1. individual factors 2. institutional factors, and 3. systemic factors | Knowledge and skill of NAFLD management  Confidence with NAFLD management in community  Acceptance of community screening  Factors within the practice management that were enablers/barriers to meeting needs of LOCATE study  Experience of the communication, referral, triage workflow and waiting times associated with the study. |  |  |
